# Microbiology of Fermented Foods and Beverages

**DOI:** 10.3390/foods9111660

**Published:** 2020-11-13

**Authors:** Theodoros Varzakas

**Affiliations:** Department of Food Science and Technology, University of the Peloponnese, Antikalamos, 24100 Kalamata, Greece; theovarzakas@yahoo.gr or t.varzakas@uop.gr

**Keywords:** fermented foods, microbiology, microbiome, health

Fermented foods are consumed all over the world and show increasing trends. They play many roles, from preservation to food security, improved nutrition and social well-being. Different microorganisms are involved in the fermentation process and the diversity of the microbiome is high.

Varzakas et al. [1] have reported on the different fermented vegetables worldwide and the versatility of microorganisms involved. They highlighted soybean tempe and other soybean paste products, sauerkraut, fermented olives, fermented cucumber and kimchi. Moreover, salting procedures are well explained along with the role of lactic acid bacteria in fermented vegetables.

One of these types of microorganisms involved in fermented foods is lactic acid bacteria, which has a strong antibacterial effect due to the production of bacteriocins [2].

Zabat et al. [3] utilized 16S rRNA amplicon sequencing to profile the microbial community of naturally fermented sauerkraut throughout the fermentation process and analyzed the bacterial communities of the starting ingredients and the production environment. They showed that the sauerkraut microbiome is rapidly established after fermentation begins and that the community is stable through fermentation and packaging for commercial sale.

On the other hand, yeasts such as *Saccharomyces cerevisiae* have been added in the dough of bakery products to improve organoleptic properties and reduce spoilage. In this direction, the potential use of *L. plantarum* UFG 121 in the biomass of the dough has been explored, as a biocontrol agent in bread production and a species- or strain-dependent sensitivity of the molds was suggested to the same microbial-based control strategy [4]. Moreover, Kara Ali [5] studied the production of the biomass of *S. cerevisiae* on an optimized medium using date extract as the only carbon source in order to obtain a good yield of the biomass. The biomass production was carried out according to the central composite experimental design (CCD) as a response surface methodology.

Finally, Bell et al. [6,7], highlighted the role of fermented foods and beverages on gut microbiota and debated for the need of transdisciplinary fields of One Health to enhance communication. They addressed nutritional and health attributes and reported that they are not included globally in world food guidelines. They also referred to some traditional African fermented products.

Fermented foods have well-known uses in human health and could help in the prevention of chronic diseases from the general gut health, to immune support, skin health, cholesterol control and lactose intolerance. More research is required in the direction of consumption of fermented foods, their benefits and daily administration.
